# Effect of host telomerase inhibition on human cytomegalovirus

**DOI:** 10.1128/jvi.01578-24

**Published:** 2025-02-05

**Authors:** Chloe M. Cavanaugh, Cora N. Betsinger, Nicole Katchur, Sherry Zhang, Karen Yang, Maciej Nogalski, Ileana M. Cristea, Daniel Notterman

**Affiliations:** 1 Department of Molecular Biology, Princeton University 200547 , Princeton, New Jersey, USA; The University of Arizona, Tucson, Arizona, USA

**Keywords:** telomerase, human herpesviruses, cytomegalovirus

## Abstract

Treatment options remain limited for human cytomegalovirus (HCMV). Host telomerase has been implicated in the pathogenesis and oncogenesis of multiple herpesviruses, most recently including HCMV. In this study, we investigated the effect of telomerase inhibition on HCMV replication, as well as the mechanism of the interaction between HCMV and host telomerase *in vitro*. We found that lytic HCMV infection increases host telomerase activity, at least in part, through modulation of hTERT expression during earlier phases of the HCMV replication cycle. We found telomerase inhibition strongly reduced viral titer for two HCMV strains in a dose-specific manner. Both post-translational pharmaceutical telomerase inhibition and siRNA-mediated knockdown of hTERT reduce HCMV yield. Telomerase inhibition results in both reduction of viral gene and protein expression across the HCMV replication cycle, and suppressed viral genome replication and viral infectivity, suggesting interference with at least early steps of the HCMV viral life cycle. Altogether, our findings indicate telomerase plays an important, perhaps non-canonical role in lytic HCMV infection which includes the support of viral replication and infectivity.

**IMPORTANCE:**

Human cytomegalovirus (HCMV) seroprevalence and morbidity in immunocompromised patients and neonates infected *in utero* remain high globally. Host telomerase has been implicated in the success of multiple infection-induced pathologies, including the success of both lytic infection and oncogenesis in certain herpesviruses. The results of this study suggest a similar biologically important role for host telomerase in lytic HCMV infection. Furthermore, these results may provide the potential for a novel, adjunctive anti-viral treatment for HCMV infection as well as insight into the viral products likely to be involved with HCMV regulation of telomerase.

## INTRODUCTION

Human cytomegalovirus (HCMV) is a double-stranded DNA herpesvirus with acute and chronic pathogenicity and potential effects in cancer progression, for which treatment options remain limited (1, 2). While anti-viral drugs attenuate active infection, their efficacy is limited and accompanied by cytotoxic effects, and viral resistance is significant (3). HCMV infection is characterized by three stages of viral gene expression: immediate early (IE), early (E), and late (L). These proteins embody a large series of activities to modulate the cellular environment and continue viral propagation (4, 5). HCMV seroprevalence and morbidity in immunocompromised patients and neonates infected *in utero* remains high in both developed and developing nations, emphasizing the need for a vaccine or permanent effective antiviral therapy (3, 6).

The reverse transcriptase, telomerase, has been implicated in herpesvirus infections (7–10). Telomerase is a key component of the eukaryotic system to manage the end replication problem and to protect the integrity of chromosome ends. Telomerase is a holoenzyme with a catalytic component, hTERT, and RNA template, hTERC (11). Demonstrated roles for host telomerase in several herpesviruses include the promotion of lytic infection, tumorigenesis, and latency (7–10). The genome of the oncogenic human herpesvirus Epstein-Barr virus (EBV) integrates close to the host hTERT gene locus and activates hTERT, the catalytic component of telomerase, to promote tumorigenesis and preserve EBV latency (10, 12). Inhibition of hTERT results in the death of EBV-infected cells and sensitizes EBV-positive tumor cells to chemotherapeutic and anti-viral therapies (10). Human herpesvirus-1 (HSV1) increases telomerase activity, and HSV1 viral titer, replication, and gene products are reduced following treatment with pharmaceutical telomerase inhibitor MST-312 (7). Recently, host cell telomerase has been shown to be sharply upregulated in response to infection by HCMV laboratory and clinical strains in human diploid fibroblast cells, which display minimal or no hTERT expression or telomerase activity at baseline (4).

Here, we sought to further characterize the role of host telomerase in lytic HCMV infection and understand how telomerase inhibition affected this relationship. We confirmed that infection with different strains of HCMV across two cell lines significantly increases telomerase activity. Moreover, viral replication is reduced following treatment with post-translational telomerase inhibitors as well as by genetic inhibition of hTERT. Our findings indicate a biologically significant relationship between HCMV and host telomerase and an important role for telomerase in the viral life cycle. Continued research into this newly established relationship between host telomerase and HCMV may provide insight regarding improved HCMV therapy and a deeper understanding of herpesvirus biology.

## MATERIALS AND METHODS

### Cells

Human lung fibroblasts (MRC-5), human foreskin fibroblasts (HFF), and human embryonic kidney (HEK293) cells were obtained from the American Type Culture Collection (ATCC). All cells were cultured in Dulbecco’s modified Eagle’s medium (DMEM) supplemented by 10% fetal bovine serum (10% FBS; BenchMark), 1× GlutaMAX (Gibco), 1× modified Eagle’s medium with non-essential amino acids (MEM NEAA; Gibco), 1× sodium pyruvate (Gibco), and 1% penicillin/streptomycin (penstrep, Thermo Fisher) (DMEM—FBS+++) at 37°C and 5% CO_2_. HFF cells were used at passages 9–12, MRC-5 cells at passages 18–22.

### Viruses

A GFP-tagged virus derived from clinical isolates, TB40/E-GFP (TB40E), and GFP-tagged laboratory strain AD169-GFP (AD169) were kindly provided by T. Shenk (Princeton University). P0 stock of AD169 and TB40E were grown in HFF and MRC-5 cells, respectively, purified by centrifugation through a sorbitol cushion (20% sorbitol, 50 mM Tris-HCl, 1 mM MgCl_2_, pH 7.2), and then concentrated and resuspended in DMEM. Viral titers were determined using a fluorescent focus assay (13, 14) on HFF or MRC-5 cells.

### Viral infection

For experiments not utilizing siRNA constructs, cells were serum starved in Dulbecco’s modified Eagle’s medium (DMEM), 1% penicillin/streptomycin (Pen/Strep, ThermoFisher) for 48 hours prior to infection. Cells were infected at a multiplicity of infection (MOI) of 3 unless otherwise specified, then incubated at 37°C for 2 hours, after which inoculum was removed, cells were washed with phosphate-buffered saline (PBS, Gibco) and collected at indicated timepoints post-infection.

### UV irradiation

UV-inactivation of TB40E virions was performed by UV irradiation of viral inoculum using Auto Cross Link settings of 600J for 3 minutes (UV Stratalinker 2400; San Diego, CA). Cell lysates were harvested at 96 hpi.

### Pharmaceutical applications

Ganciclovir (Sigma-Aldrich) was dissolved in DMSO (Sigma-Aldrich) and used at 50 µM concentration. BIBR-1532 was dissolved in DMSO and used at 15 µM and 20 µM in HFF and MRC-5 cells, respectively. MST-312 was dissolved in DMSO and used at 0.25 µM and 0.5 µM in HFF and MRC-5 cells, respectively. Non-cytotoxic doses of BIBR-1532 and MST-312 were determined for each cell type by dose titration and MTS absorbance assay (Promega), per manufacturer instructions. Unless otherwise specified, dosing for all drugs was done 2 hours prior to and post infection, with appropriate PBS washes, and at 48 hours post infection (hpi). Supernatant, RNA, and cell lysates were harvested at 96 hpi, unless otherwise specified.

### SiRNA constructs and application

For experiments using siRNA constructs, cells were transfected with siRNA against hTERT (Horizon Discovery, cat.#M-003547-02-0010) or non-coding siRNA (Horizon Discovery, cat.#D-001206-14-20) in Lipofectamine RNAiMax Reagent (Thermo Fisher) and Opti-MEM media (Gibco) 24 hours prior to infection, in media as previously described excluding Pen/Strep. Infection proceeded as previously detailed, with appropriate PBS washes, and cells were transfected again at 24 hpi. Supernatant, RNA, and cell lysates were harvested at 48–72 hpi.

### Fluorescent focus assay

When indicated, experimental HCMV viral titers were determined by assaying for IE1-positive cells on reporter plates. Harvested supernatant was serially diluted 10-fold and applied to serum-starved 96-well reporter plates, which were fixed in methanol after 24 hours and stained with mouse anti-HCMV IE1 (1B12, 1:40 dilution, provided by Shenk Lab, Princeton University) and goat anti-mouse Alexa Fluor-488 conjugated secondary antibody (1:1,000 dilution; Invitrogen, cat.#A11029). Cells were visualized, and the percentage of viral antigen-positive cells was calculated from at least 20 fields of view using the Operetta High Content Imaging System (PerkinElmer).

### RNA isolation, cDNA synthesis, and qPCR

RNA was harvested via Qiazol (Qiagen) lysis at 96 HPI unless otherwise specified. RNA isolation was performed using miRNeasy Mini Kit (Qiagen, cat.#217004), per manufacturer’s instructions. Isolated RNA samples were treated with TURBO DNA-free Kit (ThermoFisher) according to the manufacturer’s protocol. RNA concentrations were measured by NanodropOne Spectrophotometer (Thermo Scientific).

cDNA synthesis was performed with Superscript III Reverse Transcriptase (Thermo Fisher, cat.#18080044), per manufacturer’s instructions and using random hexamers (IDT), dNTPs (New England Biolabs), RNaseOUT Recombinant Ribonuclease Inhibitor (Thermo Fisher), and Mastercycler Nexus (Eppendorf). For each sample, a 17 µL solution of PCR-grade water, 1,000 ng RNA, 1 µL random primers, and 1 µL 10 µM dNTP mix was made for each sample. Samples were heated at 65°C for 5 minutes and incubated on ice for 2 minutes before adding to each: 5 µL of 5× First Strand Buffer (Invitrogen), 1 µL of 0.1 M dithiothreitol (Invitrogen), 1 µL of SuperScript III RT, and 1 µL of RNaseOUT Recombinant RNase Inhibitor (Invitrogen), for a final volume of 25 µL. For the negative controls, PCR-grade water was added instead of SuperScript III RT. Samples were then incubated at 25°C for 5 minutes and 50°C for 1 hour. The reaction was inactivated by heating the samples to 70°C for 15 minutes.

Host RNA transcripts were measured using Taqman Fast Advanced Master Mix (Thermo Fisher, cat.#4444556), per manufacturer’s instructions. Primers were purchased from Thermo Fisher (hTERT: Hs00972650_m1; hTERC: Hs03454202_s1; GAPDH: Hs02786624_g1). qPCR was performed in triplicate on Quantstudio 6 Flex (Thermo Fisher) at thermocycling conditions of 50°C for 2 minutes and 95°C for 2 minutes, and 40 cycles of 95°C for 1 second and 60°C for 20 seconds.

Viral RNA transcripts were measured using PowerUp SYBR Green Master Mix (Thermo Fisher, cat.#A25742). Primers were purchased from IDT (Table 1). qPCR was performed in triplicate on Quantstudio 6 Flex (Thermo Fisher) at thermocycling conditions of 50°C for 2 minutes and 95°C for 10 minutes, and 40 cycles of 95°C for 15 seconds and 60°C for 1 minute.

**TABLE 1:** Primer sequences for HCMV viral gene expression assessments

Primer name	Primer sequence
*UL122-F*	5′-GCGTGGAGCCTCAAAGAATTGC-3′
*UL122-R*	5′-CAGCGTGGATGATCATGTTGCG-3′
*UL123-F*	5′-ACTCAGCCTTCCCTAAGACCAC-3′
*UL123-R*	5′-AGGAGAGCACTGAGGCAAGTTC-3′
*UL54-F*	5′-TGCTTTCGTCGGTGCTCTCTAAG-3′
*UL54-R*	5′-TGTGCGGCAGGTTAGATTGACG-3′
*UL26-F*	5′-CCAGCAGCTTCCAGTATTC-3′
*UL26-R*	5′-ACCTGGATCTGCCCTCATC-3′
*UL83-F*	5′- TGGCTACGGTTCAGGGTCAGAATC-3′
*UL83-R*	5′-AGATGCGGTAGATGTCGTTGGC-3′
*UL99-F*	5′-ACGACAACATCCCTCCGACTTC-3′
*UL99-R*	5′-TCTGTTGCCGCTCCTCGTTATC-3′

### Assessment of telomerase activity

Telomerase activity was assayed using a TRAPeze Telomerase Detection Kit (MilliporeSigma, cat.#S7700), following manufacturer instructions. Cells were harvested by scraping in cold PBS at 96 hpi unless otherwise specified. Cells were lysed in a solution of 1× CHAPS Lysis Buffer (MilliporeSigma), resuspended Protease Inhibitor Cocktail Tablets (Roche), and RNaseOUT Recombinant RNase Inhibitor (Invitrogen) for 40 minutes. The lysate was next centrifuged at 4°C and 16,000 × *g* for 20 minutes. Lysate protein concentration was measured with Pierce 660 nm Protein Assay (Thermo Fisher) and SpectraMax GeminiXS (Molecular Devices Corporation) per manufacturer’s instructions. A master mix of 18.8 µL PCR-grade water, 2.5 µL 10× TRAP Reaction Buffer (Sigma-Aldrich), 0.5 µL 50× dNTP Mix (Sigma-Aldrich), 0.5 µL TS Primer (Sigma-Aldrich), 0.5 µL TRAP Primer Mix (Sigma-Aldrich), and 0.2 µL OneTaq Hot Start DNA Polymerase (New England Biolabs) per normalized sample was prepared for final reaction volume of 25 µL. PCR was performed with Mastercycler Nexus (Eppendorf) with thermocycling conditions of 30°C for 45 minutes, by 95°C for 5 minutes, 35 cycles of 95°C for 30 seconds, 52°C for 30 seconds, 72°C for 1 minute, and, finally, 72°C for 3 minutes. Positive controls included HEK293 cells and TSR8 TRAPeze internal positive control. Negative controls included CHAPs lysis buffer, control with no added polymerase, and heat-inactivated samples. Heat inactivation for negative samples was performed by incubation at approximately 80–90°C for 20 minutes. Samples were subsequently run on 10% non-denaturing polyacrylamide gel in 0.5× Tris-Borate-EDTA buffer. For polymerization, 0.5 µL of *N,N,N′,N′*-tetramethylethylenediamine (VWR) and 10 µL of 10% ammonium persulfate (Fisher Scientific) were added per 1 mL of polyacrylamide gel solution. A 25 kb DNA ladder was used for reference (Invitrogen cat.# 10597-011). Gels were run at 30 mA/170 V for 3 hours and then stained with GelRed Nucleic Acid Stain 10,000× Water (Sigma-Aldrich) for 30 minutes before imaging with ChemiDoc MP Imaging System (BioRad). Quantification was performed on Image Lab Software (BioRad). Following band quantification, relative telomerase activity was calculated as (∑sample band quantification/sample internal control band quantification)/∑HEK293 band quantification/HEK293 internal control band quantification).

### Western blot

Cell lysate was resuspended in 2× Laemmli Sample Buffer (BioRad) and PBS in a 1:1 ratio and homogenized. Samples were incubated at 70°C for 5 minutes, and protein concentration was measured with Pierce 660 nm Protein Assay (ThermoFisher) with ionic detergent compatibility reagent (IDCR; ThermoFisher), according to manufacturer’s instructions, using the SpectraMax GeminiXS (Molecular Devices Corporation). For gel electrophoresis, an SDS-PAGE gel (Any kD Mini-PROTEAN TGX Precast Protein Gels; BioRad) was loaded with 8 µL of Precision Plus Protein Standards (BioRad) and 10 µL of normalized sample. Gels were run at 80 V for 2 hours at RT in 1× Tris/Glycine/SDS Buffer (BioRad), and then proteins were transferred onto a methanol-wetted Millipore Immobilon-P PVDF membrane at 120 mA at 4°C overnight using two black transfer pads and two pieces of Whatman paper soaked in Tris-glycine transfer buffer (BioRad). Membranes were rinsed with 10× Tris-buffered saline (TBS; BioRad) and blocked for 1 hour at RT with 5% nonfat dry milk and 1× Tris-buffered Saline/Triton (BioRad).

Primary antibodies were mouse monoclonal antibodies anti-IE1 (1B12; 1:500 dilution), anti-IE2 (3A9; 1:500 dilution), anti-pUL26 (7H1-5; 1:100 dilution), anti-pUL69 (10E11; 1:100 dilution), anti-pUL99 (10B4-29; 1:100 dilution), all provided by T. Shenk (Princeton University), and anti-β-actin-HRP (1:100,000 dilution; Abcam; cat.# ab49900). Goat anti-mouse antibody (1:10,000 dilution; Jackson ImmunoResearch Laboratories Inc.; cat.# 115-035 003) conjugated with horseradish peroxidase was used as the secondary antibody. HyBlot CL autoradiography film (Thomas Scientific) was used to manually develop the blot before imaging with ChemiDoc MP Imaging System (BioRad). Quantification was performed on Image Lab Software (BioRad).

### Parallel reaction monitoring-based quantification of HCMV protein abundances

#### Sample preparation for MS analysis

To analyze viral protein abundances during infection, cells were harvested at 24, 48, 72, and 96 hpi by washing and scraping in cold PBS. Cells were collected by centrifugation, washed twice with cold PBS, snap frozen in liquid nitrogen, and stored at −80°C. To lyse, frozen cell pellets were briefly thawed on ice and resuspended in 5% SDS, 25 mM TCEP (Thermo Fisher, 77720), and 50 mM chloroacetamide. Lysis was accomplished using repeated rounds of boiling followed by cup horn sonication and proteins were recovered using methanol-chloroform precipitation (15). Samples were resuspended in 100 mM HEPES (pH 8.2) at a 0.5 µg/µL protein concentration (as determined by BCA assay) and digested overnight at 37°C with MS-grade Pierce trypsin (Thermo Fisher 90057) at a 1:50 trypsin:protein mass ratio. Following overnight digestion, samples were acidified to 1% trifluoroacetic acid and desalted using 3M Empore C18 Extraction Disks (Thermo Fisher 14-386-2). Peptides were concentrated to near dryness by vacuum centrifugation and resuspended in 1% FA/1% ACN for LC-MS/MS analysis.

#### Targeted MS analysis by PRM

Targeted MS analysis was performed via LC-MS/MS using a Dionex Ultimate 3000 nanoRSLC coupled to a Q Exactive HF mass spectrometer (Thermo Fisher) (15). Peptides were separated by reverse-phase chromatography on an EASY-Spray HPLC Column (Thermo Fisher, 500 mm length, 2 µm particle size, 75 µm diameter) using a 60 minute gradient (3%–35% B) with 250 nL/min flow rate. Mobile phase A consisted of 0.1% formic acid in water and mobile phase B consisted of 0.1% formic acid in 97% ACN. The PRM method consisted of targeted MS2 scans recorded in profile mode performed at a resolution of 30,000, with an AGC target of 1e5, maximum inject time of 200 ms, isolation window of 1.2, and normalized collision energy of 27% controlled by a peptide inclusion list derived from the TRUSTED targeted MS assay (15). An MS1 scan was performed at a resolution of 15,000 across a mass range of 400–2,000 with an automatic gain control (AGC) of 3e6, and max injection time of 15 ms.

#### Quantification and analysis of HCMV protein abundances

Label-free quantitation was performed using previously described methods (15) and Skyline Daily software for targeted proteomics. A summed area under the curve of three transitions per peptide was used for quantitation. MS1 intensity-based normalization, with peak intensity determined using RawMeat (Vast Scientific), was performed for normalization across samples and replicates. Individual peptides were normalized to their average abundance across all time points/sample conditions. Following normalization, if there were multiple peptides per protein, they were averaged. Average protein abundance was then calculated across replicates, and proteins were organized by temporality (15). Full data are available at https://panoramaweb.org/telomerasehcmvprm.url with ProteomeXchange ID PXD058198.

### Extracellular genome extraction

Cell growth media was harvested at 96 hpi, subjected to free DNA digestion, and then subjected to phase extraction with 1:1 phenol:chloroform (VWR). Linear acrylamide (Invitrogen), 3M NaOAc, and 100% EtOH were added to the extracted phase layer and precipitated at −20°C overnight. Following centrifugation and supernatant removal, samples were treated with 70% EtOH and centrifuged, and the supernatant was removed. The resulting pellet was dried with Speedvac and resuspended in RNAse free water for qPCR analysis.

### Intracellular genome extraction

Cell lysates were harvested at 96 hpi, supernatant extracted following sonication, treated with Resuspensions Buffer [400 mM NaCl, 10 mM Tris (pH 7.5–8), 10 mM EDTA, MilliQ water], then Proteinase K (Sigma Aldrich) and 10% SDS, and incubated at 37°C overnight. Samples were then subjected to phase extraction with 1:1 phenol:chloroform (VWR). Linear acrylamide (Invitrogen), 3M NaOAc, and 100% EtOH were added to the extracted phase layer and precipitated at −20°C overnight. Following centrifugation and supernatant removal, samples were treated with 70% EtOH, centrifuged, and the supernatant removed. The resulting pellet was dried with Speedvac and resuspended in RNAse free water for qPCR analysis.

### Statistical analyses

Statistical significance was analyzed using either unpaired *t*-test for comparison between two groups or one-way ANOVA with Tukey test for comparison between three and more groups by the GraphPad Prism 10 software. *P* < 0.05 was considered significant. Graphs were produced using the GraphPad Prism version 10 software.

## RESULTS

### HCMV infection increases telomerase activity and hTERT expression in a dose- and time-dependent manner

**Fig 1:**
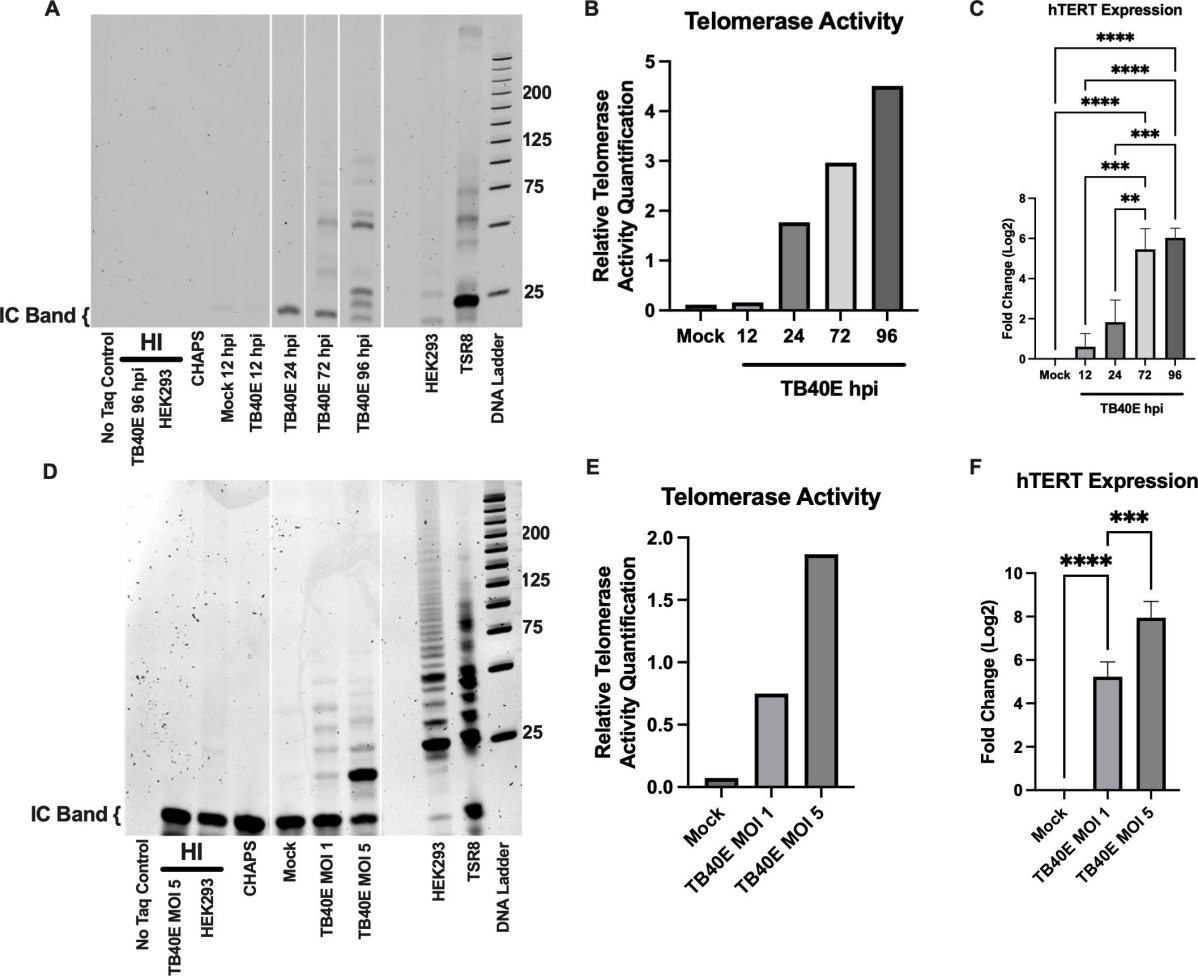
HCMV infection increases hTERT expression and telomerase activity in a dose- and time-dependent manner. (A and B). MRC5 cells were infected with TB40E at MOI 5 and harvested at 12, 24, 72, and 96 hpi. (A) Telomerase repeat amplification protocol (TRAP) assay of protein extracts prepared from mock-infected or HCMV TB40E-infected MRC5 cells at 12, 24, 72, and 96 hpi. IC Band—internal control band. No Taq control = negative control, HI = heat inactivated negative control, CHAPS = negative control, HEK293 = positive control, TSR8 = positive control. DNA ladder 25 bp increments. White vertical spaces indicate where the gel image was spliced to remove lanes left intentionally blank for clarity. (B) Quantification of TRAP-determined telomerase activity in (A), one replicate. (C) MRC5 cells were infected with TB40E at MOI 3 and harvested at 12, 24, 72, and 96 hpi. Quantification of hTERT expression. ANOVA with Tukey. **P = 0.0013, ***P = 0.0004, ****P < 0.0001. Error bars indicate standard deviation. Three replicates. (D–F). MRC5 cells were infected with HCMV strain TB40E at MOI 1 or 5 and harvested at 72 hpi. (D) TRAP assay of protein extracts prepared from mock-infected or HCMV TB40E-infected MRC5 cells, 72 hpi. DNA ladder 25 bp increments. White vertical spaces indicate where gel image was spliced to remove lanes left intentionally blank for clarity. (E) Quantification of TRAP-determined telomerase activity in (D), one replicate. (**F**) Quantification of hTERT expression. ANOVA with Tukey. Error bars indicate standard deviation. ***P = 0.0003, ****P < 0.0001. Four replicates.

To confirm the effect of HCMV infection on telomerase activity and provide new insight into hTERT expression following CMV infection, we infected fibroblasts (MRC5 cells) with the low-passage HCMV strain TB40E (16). hTERT expression was measured with qPCR, and telomerase activity was assessed using a TRAP assay (17). hTERT expression and telomerase activity increased throughout the HCMV replication cycle (Fig. 1A through C), and these increases were dependent on the input multiplicity of infection (Fig. 1D through F). These observations indicate that the HCMV-mediated increase in telomerase activity is, at least in part, mediated through elevated hTERT expression.

### HCMV transcription, but not late gene expression, is necessary for increased telomerase activity

**Fig 2:**
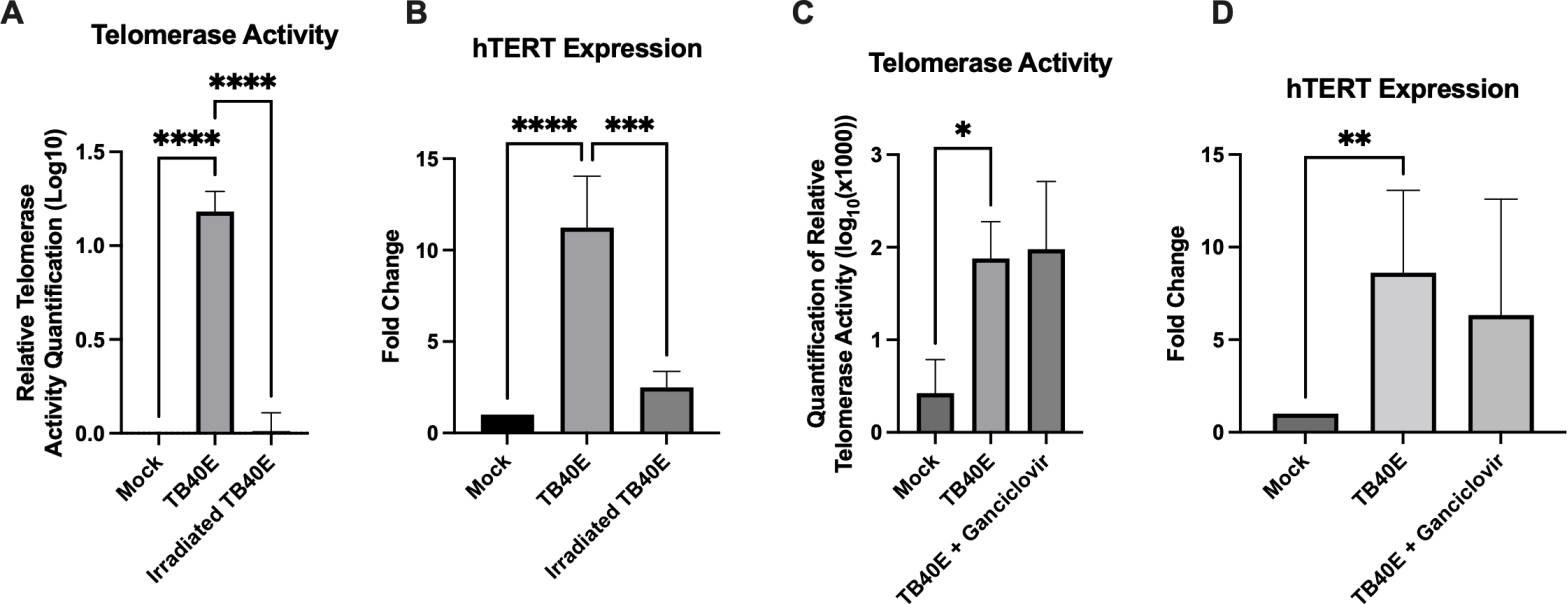
Viral transcription, but not late gene expression, is important for HCMV-mediated telomerase activity. (A and B). Infection of MRC5 cells with TB40E, or UV-irradiated TB40E, MOI 3, harvested 96 hpi. (A) Quantification of telomerase activity. *P* < 0.0001. Three biological replicates. (B) Quantification of hTERT expression. ***P = 0.0001, ****P < 0.0001. Calculations relative to Mock. Four biological replicates. (C and D) Treatment of TB40E-infected MRC5 cells, MOI 3, with 50 µM ganciclovir. (C) Quantification of telomerase activity. *P* = 0.0189. Three biological replicates. (D) Quantification of hTERT expression. *P* = 0.0107. Calculations relative to Mock. Four biological replicates. All results were determined by ANOVA statistical analyses with Tukey. All error bars represent standard deviation.

Next, infection conditions were altered to ascertain which stages of the viral life cycle are required for induction of HCMV-mediated telomerase activity. Irradiation of TB40E prior to attempted infection of MRC5 cells abrogated the induction of telomerase activity and hTERT expression along with viral gene expression (Fig. 2A and B; Fig. S1A through G). This finding suggests newly expressed viral genes are important for telomerase activity, rather than non-specific interaction between viral structures and the host cell or effect of tegument proteins. Post-infection treatment with ganciclovir, a nucleoside analog that affects late gene production and is used to attenuate active HCMV infection clinically (3, 18) dramatically reduced viral titer (Fig. S1b) but did not affect telomerase activity or hTERT expression (Fig. 2C and D), suggesting that genes expressed during the immediate early and early stages of the life cycle are sufficient for inducing telomerase activity.

### Telomerase inhibition reduces HCMV yield, RNA, and protein levels

**Fig 3:**
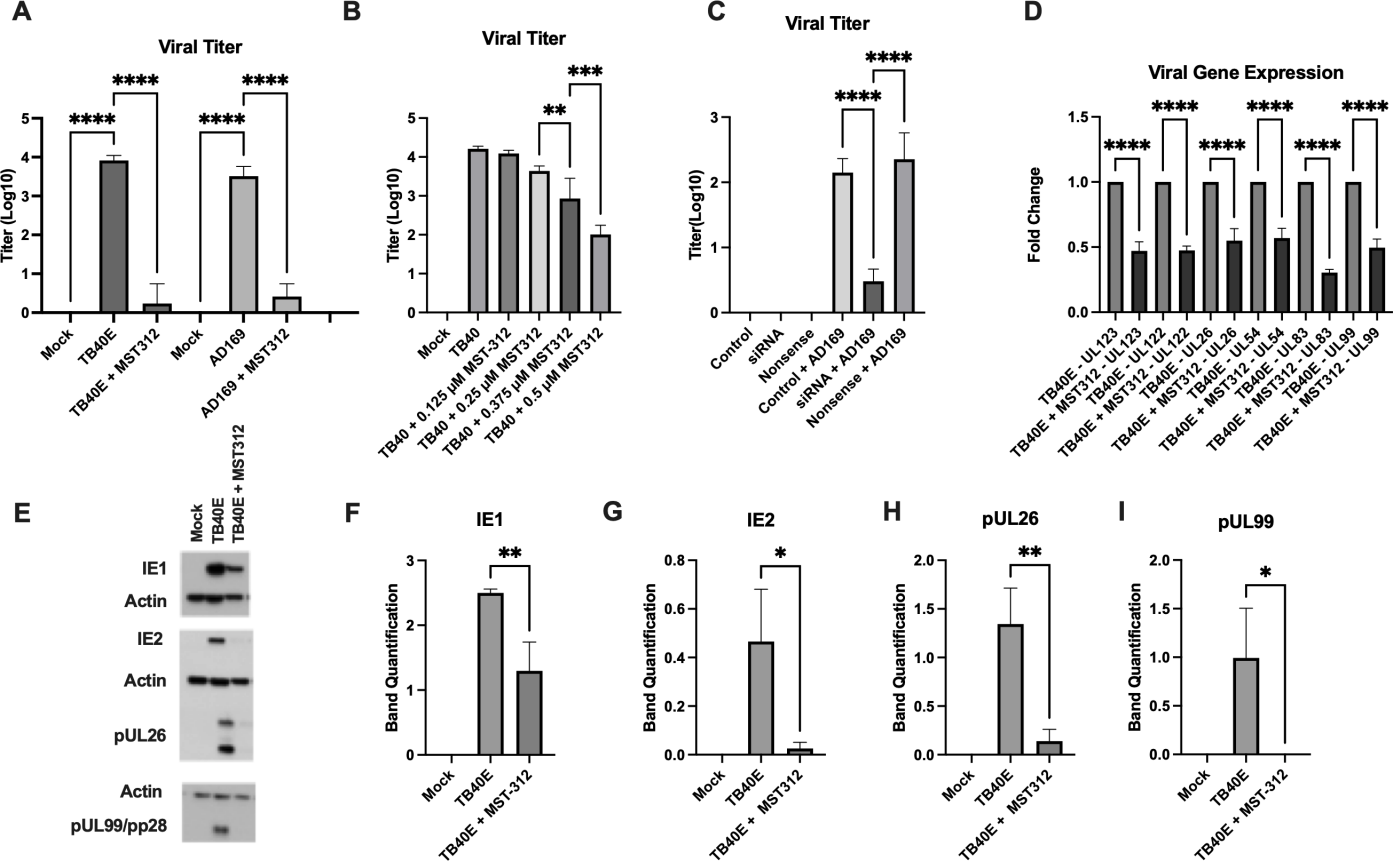
Telomerase inhibition sharply reduces HCMV replication, gene expression, and protein levels. (**A and B**) MRC5 or HFF cells were infected with HCMV strain TB40E or AD169 at MOI 3, respectively, treated with 0.5 µM or 0.25 µM MST-312, respectively, and harvested at 96 hpi. ANOVA statistical analyses with Tukey. All error bars represent standard deviation. (**A**) Quantification of TB40E and AD169 viral titer following treatment with MST312 hTERT inhibitor. *P* < 0.0001. Four biological replicates. (**B**) Quantification of TB40E viral titer. **P = 0.0075 and ***P = 0.0005. Four biological replicates. (**C**) Control = HFF cells. siRNA = HFF cells treated with siRNA targeting hTERT. Nonsense = HFF cells treated with non-targeting siRNA. Control + A.D.169169 = A.D.169169-infected HFF cells. hTERT + A.D.169169 = A.D.169169-infected HFF cells treated with siRNA targeting hTERT. Nonsense + A.D.169169 = A.D.169169-infected HFF cells treated with non-targeting siRNA. All infected arms received AD169 MOI 3. Quantification of viral titer. *P* < 0.0001. ANOVA statistical analysis with Tukey. Error bars represent standard deviation. Four biological replicates. (**D–I**) MRC5 cells were infected with HCMV strain TB40E at MOI 3, treated with 0.5 µM MST-312, respectively, and harvested at 96 hpi. ANOVA statistical analyses with Tukey. All error bars represent standard deviation. (**D**) Viral gene expression following MST-312 treatment. *P* < 0.0001. Six biological replicates. (**E**) Representative western blots of immediate early proteins IE1 and IE2, early protein pUL26, and late protein pUL99/pp28 with actin control. (**F**) Relative quantification of immediate early protein IE1. *P* < 0.0001. Three biological replicates. (**G**) Relative quantification of immediate early protein IE2. *P* = 0.0065. Three biological replicates. (**H**) Relative quantification of early protein pUL26. *P* = 0.0006. Three biological replicates. (**I**) Relative quantification of late protein pUL99/pp28. *P* = 0.0093. Three biological replicates.

To assess the effect of telomerase inhibition on viral replication, TB40E-infected fibroblasts were treated with non-cytotoxic doses (Fig. S2) of pharmaceutical inhibitor MST-312, a synthetic analog of epigallocatechin gallate (EGCG) that competitively inhibits telomerase (7). MST-312 inhibition resulted in a significant and dose-dependent reduction of viral titer as measured by fluorescent focus assay (FFA) (Fig. 3A and B) with significant reduction of telomerase activity (Fig. S3A and B). hTERT expression was not affected by MST-312 inhibition (Fig. S3D and 4F) though, unexpectedly, hTERC expression was increased in a dose-dependent fashion (Fig. S3E and G) indicating the existence of post-translational hTERC regulation not previously characterized. To further assess whether the effect of pharmaceutical telomerase inhibition is a consistent effect for HCMV infection, we assessed infection with another HCMV strain, the laboratory-adapted AD169 strain (19). Indeed, MST-312 treatment of AD169-infected fibroblasts (HFF cells) recapitulated the results observed for TB40E (Fig. 3A). As an additional confirmation, the assays were repeated with a second telomerase inhibitor, BIBR-1532 (20). Results were consistent though the effects were more subtle (Fig. S4). BIBR-1532 non-competitively inhibits telomerase by interrupting enzyme processivity (20). To further demonstrate that the observed reduction in viral titer with pharmaceutical inhibition was due specifically to telomerase inhibition, and not an “off-target” effect, an siRNA construct targeting hTERT was applied 24 hours prior to, and again 24 hours following, AD169 infection of HFF cells. At 48–72 hpi, viral titer was significantly reduced (Fig. 3C), and both hTERT expression and telomerase activity were confirmed to be significantly attenuated (Fig. S3H through J). These findings suggest that abrogation of telomerase activity *per se* is likely to be responsible for the observed reduction in viral titer and supports the conclusion that hTERT expression and activity are important for HCMV-mediated telomerase activity.

To characterize the effect of telomerase inhibition on HCMV gene expression, qPCR and western blot were used to assess viral mRNA and protein expression. Telomerase inhibition significantly reduced mRNA expression of viral genes from each phase of the HCMV replication cycle (Fig. 3D). Consistent with the effect on mRNA expression, western blot analysis showed a significant reduction in the levels of representative viral proteins expressed during each of the different phases of the HCMV life cycle following treatment of infected cells with either pharmaceutical telomerase inhibition or siRNA knockdown against hTERT (Fig. 3E through I; Fig. S3K and L).

**Fig 4:**
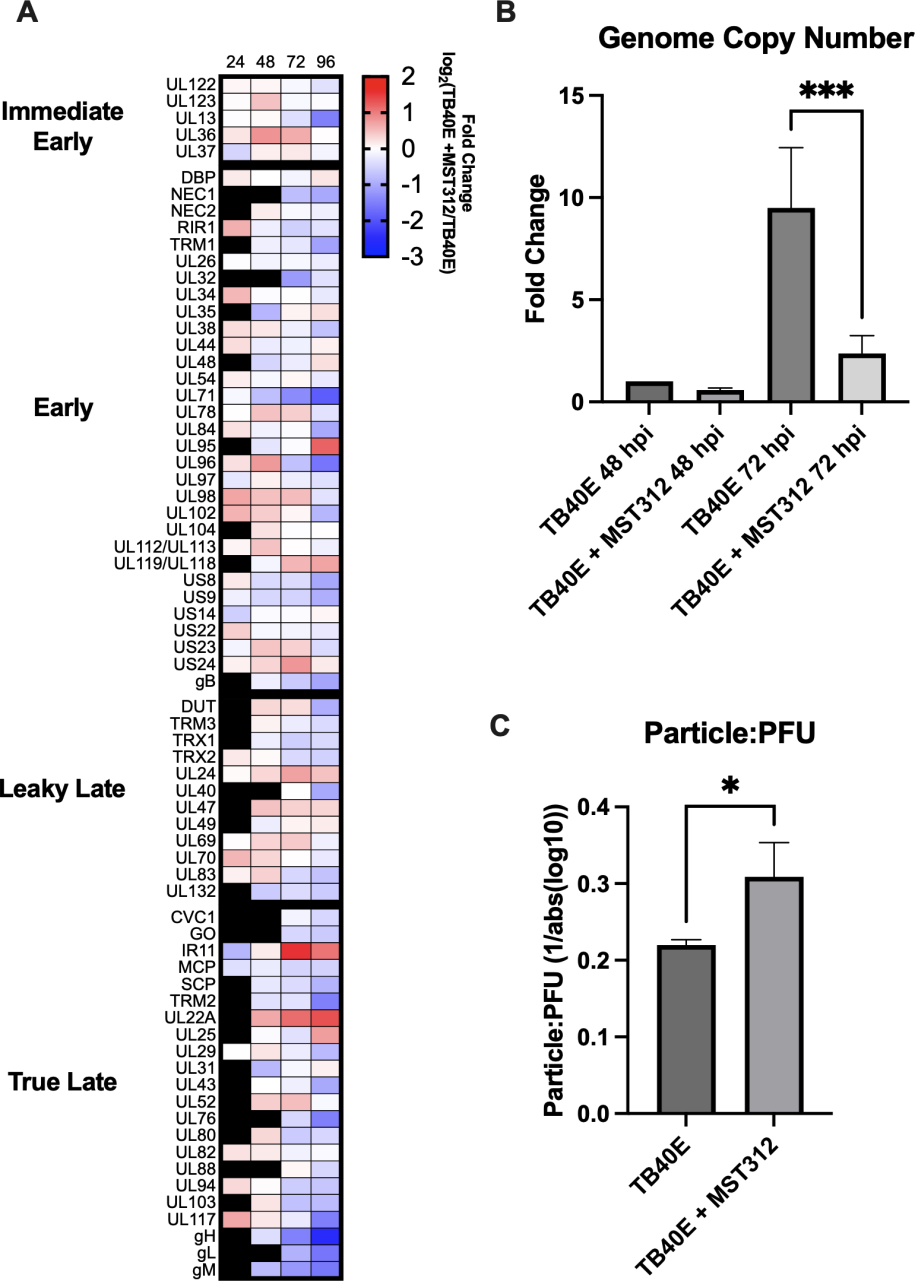
Telomerase is necessary for effective viral replication and infectivity. MRC5 cells were infected with TB40E (MOI 3) and treated with pharmaceutical inhibitor MST-312 (0.5 µM). (**A**) Lysates analyzed by parallel reaction monitoring (PRM) to determine relative protein abundances of a pre-set protein panel. Scale indicates fold change of log_2_((TB40E + MST-312)/TB40E). Black squares = not detected. Three biological replicates. (**B**) Viral genome copy number. Fold Change (2^−(ΔΔCt)^) relative to TB40E 48 hpi. *P* = 0.0003. ANOVA with Tukey. Error bars indicate standard deviation. Three biological replicates. (**C**) PFU was quantified by fluorescent focus assay. Relative particle quantification was assessed with qPCR. *P* = 0.0271. *t*-test. Error bars indicate standard deviation. Three biological replicates.

To further assess the effect of telomerase inhibition on HCMV protein abundances, we employed a targeted mass spectrometry assay, based on parallel reaction monitoring (PRM), which was designed to specifically detect and quantify viral proteins from the different temporal classes of HCMV replication (15). The PRM assay was conducted in TB40E-infected, MST-312-treated MRC5 cell lysates at 24, 48, 72, and 96 hpi. Pharmaceutical telomerase inhibition generally resulted in reductions in HCMV protein abundances throughout all phases of the virus replication cycle (Fig. 4A). Proteins exhibiting significant reduction in abundance included pUL70, pUL102, pUL84, pUL98, pUL117, and envelope glycoproteins gM, gH, and gB among others (Fig. S5). Additionally, several proteins showed notable increases in abundance, including pUL22A, IR11, and pUL95. To assess the impact of telomerase inhibition on HCMV viral replication, viral genome copy number was assessed. Telomerase inhibition resulted in the reduction of HCMV genome copy number by 72 hpi (Fig. 4B). A particle:PFU analysis employed to assess HCMV infectivity identified markedly reduced infectivity following telomerase inhibition (Fig. 4C).

## DISCUSSION

HCMV remains a target for more effective anti-viral therapies. The observation that HCMV infection increases host telomerase activity, at least in part, due to augmented hTERT expression during earlier stages of the HCMV replication cycle, together with the finding that telomerase inhibition dramatically reduces viral titer in two HCMV strains in a dose-specific manner strongly suggests a biologically significant relationship between HCMV and host telomerase. We found that post-translational pharmaceutical telomerase inhibition and genetic knockdown of telomerase activity through an siRNA construct targeting hTERT gene expression reduce HCMV yield. To our knowledge, there is no current or investigational therapy for any virus that utilizes either post-translational telomerase inhibition or hTERT knockdown.

Our findings suggest telomerase inhibition interferes with multiple steps of the HCMV viral life cycle, as is the case for HSV (7, 21). Post-translational telomerase inhibition reduced both viral gene expression and viral protein levels throughout the HCMV life cycle. The alteration in the abundance of relevant viral proteins observed with western blot and PRM analysis, combined with the clear findings implicating viral replication (genome copy number) and infectivity lend support to the mechanistic importance of telomerase to productive HCMV infection.

The reduction in HCMV genome copy number along with the reduction in abundance of viral replication proteins following telomerase inhibition indicates that telomerase inhibition hampers viral replication. The reductions in core replication proteins pUL70 (primase), pUL102 (helicase/primase-associated factor), pUL98 (alkaline nuclease), pUL84, which facilitates initiation of lytic replication, and other replication supporting proteins including pUL117, among others (5, 22–24), suggest a broad role for telomerase in the support of viral replication. Some reduction in pUL54 (polymerase), a common target for mutations that confer resistance to existing anti-viral therapeutics (25), suggests telomerase inhibition may be useful as an adjunctive therapy.

HCMV envelope glycoproteins are important for both successful initial virion attachment and cell-based spread, mediated by cell-cell fusion events (26). gB, gM, gH, and gL are integral for effective membrane fusion and syncytium formation across multiple cell types (26–28). Furthermore, gB and gH are integral components of extracellular vesicles released by HCMV to enhance viral spread by possibly stimulating a pro-viral environment among uninfected cells (29). Impairment of extracellular vesicle biogenesis pathways has been shown to slow viral spread and reduce viral efficiency (29, 30). Overall, the reduced abundance of envelope glycoproteins, in concert with reduced particle infectivity, suggests telomerase is ultimately important to processes leading to the expressions of viral proteins needed for both effective virion attachment and cell-cell fusion.

Of the few proteins whose abundance noticeably increased following telomerase inhibition, pUL22A and RL11 represent anti-inflammatory and immune-evasive viral processes, respectively (5). It is not clear why these proteins should have increased expression in the context of telomerase inhibition, but they may represent a viral response to increased stress. Further investigation into the proteins affected by telomerase inhibition will likely provide insights on the specific mechanistic interactions between telomerase and HCMV, including the potential presence of a preceding watershed interaction responsible for the abrogation of HCMV infection by host telomerase inhibition.

Though these findings indicate that telomerase is biologically important for successful HCMV infection, they remain correlative in nature. Additional work is required to establish the specific mechanism by which HCMV increases telomerase activity. The difference in sensitivity between TB40E and AD169, which is known for acquired mutations, to treatment with BIBR1532 may also provide insight to the mechanism by which telomerase supports HCMV infection. Further *in vivo* studies in mammalian models are necessary to learn whether telomerase activity plays a role in active infection in the mammalian host. This is an essential step toward the evaluation of telomerase inhibition as a potential therapeutic avenue to combat active HCMV infection. Though the present findings were robust and significant across two cell lines and two strains of HCMV, only diploid fibroblast cell lines were used, while HCMV infects many cell types. Characterizing the significance of telomerase in human HCMV infection and in other readily infected cell types may further delineate the potential clinical significance of a telomerase-based HCMV therapy. The role of telomerase in maintaining latent HCMV infection should also be explored.

Several non-canonical functions of telomerase are paralleled by features of active HCMV infection. Both have pro-proliferative as well as pro-migratory effects on the cell (31, 32). Both improve mitochondrial capacity (32, 33). It is not entirely clear how HCMV enables the mitochondria to effectively manage the stress the virus places on the organelle by the elevation of the ETC rate during infection (33); hTERT reduces mitochondrial oxidative stress (32), which if unchecked can deplete mitochondrial DNA (mtDNA) (33). Depletion of mtDNA is known to impair HCMV viral replication (33). Telomerase has been shown to provide additional protection from DNA damage, a feature potentially advantageous to a DNA virus (32). Future mechanistic investigations may identify any of these coincidences, or others, to be roles in which telomerase aids HCMV active infection.

Overall, the present findings strongly indicate a biologically meaningful role for telomerase in HCMV active infection, specifically supporting viral replication and infectivity, and suggest the potential for a novel clinical anti-viral treatment for active HCMV infection. These findings expand our knowledge about the more general phenomenon of telomerase involvement in herpesvirus infections. Telomerase inhibition can be utilized to better study animal models of CMV as well as other herpesviruses and to gain deeper insights into HCMV and herpesvirus biology.

## Data Availability

Full data are available at https://panoramaweb.org/telomerasehcmvprm.url with ProteomeXchange ID PXD058198.

## SUPPLEMENTAL MATERIAL

Supplemental material jvi.01578-24-s0001.docx Figures S1 to S6; Tables S1 and S2.

ASM does not own the copyrights to Supplemental Material that may be linked to, or accessed through, an article. The authors have granted ASM a non-exclusive, world-wide license to publish the Supplemental Material files. Please contact the corresponding author directly for reuse.
